# Idiopathic Focal Eosinophilic Enteritis (IFEE), an Emerging Cause of Abdominal Pain in Horses: The Effect of Age, Time and Geographical Location on Risk

**DOI:** 10.1371/journal.pone.0112072

**Published:** 2014-12-02

**Authors:** Debra C. Archer, Deborah A. Costain, Chris Sherlock

**Affiliations:** 1 Institute of Infection and Global Health/School of Veterinary Science, University of Liverpool, Leahurst Campus, Neston, United Kingdom; 2 Department of Mathematics and Statistics, Lancaster University, Lancaster, United Kingdom; The University of Melbourne, Australia

## Abstract

**Background:**

Idiopathic focal eosinophilic enteritis (IFEE) is an emerging cause of abdominal pain (colic) in horses that frequently requires surgical intervention to prevent death. The epidemiology of IFEE is poorly understood and it is difficult to diagnose pre-operatively. The aetiology of this condition and methods of possible prevention are currently unknown. The aims of this study were to investigate temporal and spatial heterogeneity in IFEE risk and to ascertain the effect of horse age on risk.

**Methodology/Principal Findings:**

A retrospective, nested case-control study was undertaken using data from 85 IFEE cases and 848 randomly selected controls admitted to a UK equine hospital for exploratory laparotomy to investigate the cause of colic over a 10-year period. Generalised additive models (GAMs) were used to quantify temporal and age effects on the odds of IFEE and to provide mapped estimates of ‘residual’ risk over the study region. The relative risk of IFEE increased over the study period (p = 0.001) and a seasonal pattern was evident (p<0.01) with greatest risk of IFEE being identified between the months of July and November. IFEE risk decreased with increasing age (p<0.001) with younger (0–5 years old) horses being at greatest risk. The mapped surface estimate exhibited significantly atypical sub-regions (p<0.001) with increased IFEE risk in horses residing in the North-West of the study region.

**Conclusions/Significance:**

IFEE was found to exhibit both spatial and temporal variation in risk and is more likely to occur in younger horses. This information may help to identify horses at increased risk of IFEE, provide clues about the aetiology of this condition and to identify areas that require further research.

## Introduction

Gastrointestinal disorders that result in development of acute abdominal pain (colic) are one of the most frequent causes of death in domesticated horses and have a significant impact on equine health and welfare. Around 9% of colic episodes in the general equine population attended to by a veterinary surgeon (primary care setting) require surgical treatment or euthanasia [1], and mortality rates following surgical treatment are around 30% to hospital discharge [1], [2]. In addition, colic has economic implications for horse owners and the wider equine industry; the estimated annual cost of colic to the USA equine industry was $115.3M in 2001, with mortality accounting for 66% of this figure [3].

Equine colic is multifactorial in nature, and epidemiological studies have identified various risk factors including change in diet, exercise and stabling routines, high burdens of intestinal parasites and dental disease [4]. A number of different forms of gastrointestinal disorders are recognised as a cause of abdominal pain in horses [5], some of which require surgical management to prevent death from occurring. In contrast to humans and smaller domesticated species, the size and temperament of horses limits the extent to which diagnostic investigations, including certain imaging modalities such as computed tomography, can be utilised to determine the exact cause of abdominal pain. Some specific forms of colic have been shown to be associated with different risk factors such as bowel obstruction by a pedunculated lipoma [6], simple colonic obstruction/distention (SCOD) colic [7], epiploic foramen entrapment of small intestine [8], [9] and large intestinal volvulus [10]. This knowledge can facilitate diagnosis of these specific lesions in conjunction with the results of clinical examination and adjunctive diagnostic tests. Early identification of horses likely to require surgical management, prior to the development of severe intestinal and cardiovascular compromise, is also important in maximising survival following surgery [11]. In addition, knowledge of factors that alter the risk of colic can be used to implement management strategies for prevention and may help to generate hypotheses about disease causation that can be used as a basis for future research.

In the late 1990's reports of an unusual intestinal lesion causing colic in horses in the USA, UK and Republic of Ireland began to emerge [12]. These cases were characterised by acute onset abdominal pain and clinical findings consistent with simple (non-strangulating) obstruction of the small intestine (small bowel). Exploratory laparotomy revealed one or more visually striking hyperaemic, focal lesions within the small intestine that resulted in obstruction of ingesta and fluid at one (or occasionally several) of these sites. Histologically, these lesions were characterised by transmural infiltration dominated by eosinophils and macrophages, particularly within the muscularis layers [13], [14]. These lesions have been variously termed inflammatory bowel disease [15], idiopathic eosinophilic enteritis [16], multifocal eosinophilic enteritis [17], circumferential mural bands [18] and, more consistently, idiopathic focal eosinophilic enteritis (IFEE) [13], [19]. Since these lesions were first reported, IFEE cases have continued to occur in certain geographic regions and the number of cases anecdotally occurring in these areas appears to be increasing [13], [18].

Eosinophils are inflammatory leukocyte cells that are found in relatively small numbers within the tissues of the gastrointestinal tract in humans and animals. They play an important role in host defence and, in particular, in immunity to helminth parasites [20], [21]. Most research on disease arising as a consequence of accumulation of abnormally large numbers of eosinophils within the gastrointestinal tract has been conducted in humans. Commonly referred to as eosinophilic gastrointestinal disorders (EGID) in humans, these are an important, but relatively poorly understood group of conditions that have been increasingly recognised over the last 10–15 years [22], [23]. EGIDs are further sub-classified depending on which part of the gastrointestinal tract is affected by eosinophilic infiltration and whether they are primary in nature or are secondary to recognised causes of gastrointestinal eosinophilia such as drug reactions and parasite infestation. The aetiology of most primary EGID in humans' remains poorly understood at present but there is a strong genetic and allergic component with clinical and immunopathologic features similar to asthma [21].

Several forms of eosinophilic disease that involve the gastrointestinal tract are recognised in the horse. Multisystemic eosinophilic epitheliotrophic disease (MEED) is characterised by eosinophilic infiltration in a variety of tissues, most commonly the gastrointestinal and upper respiratory tracts and skin, although any organ can be affected [24], [25]. The aetiology of this condition is unknown and is associated with a poor prognosis, although some horses have responded to immunosuppressive treatment [26]. Idiopathic eosinophilic enterocolitis (EC) is characterised by diffuse eosinophilic infiltration confined to the gastrointestinal tract only and is a rare form of equine inflammatory bowel disease. Presenting clinical signs include recurrent colic, weight loss and low serum protein and albumin [27], [28], and some cases have responded well to oral prednisolone therapy [28]. Focal eosinophilic lesions of the small intestine and colon have been recognised secondary to localised infiltration of the bowel wall by the fungus *Pythium* sp. [29] or encapsulated nematodes [30]. In addition to IFEE, in which eosinophilic infiltration is confined the small intestine, idiopathic focal infiltration of eosinophils confined to the large colon has also been reported [31]. Horses with the latter two diseases most commonly present with clinical signs of acute abdominal pain and usually require surgical management.

The characteristic IFEE lesions seen in horses have not been identified in humans or other animal species nor do we currently know their aetiology. A potential immediate hypersensitivity or typical IgE-mediated helminth reaction has been considered unlikely due to the concurrent presence of macrophages and T lymphocytes but with an absence of mast cells [14], [32]. In a preliminary multicenter epidemiological study conducted by Archer et al. [33], nematode and cestode burdens in affected horses were demonstrated to be low and not statistically different from a random selection of normal horses, suggesting that there is no association between IFEE and large burdens of these equine gastrointestinal parasites. In the same study, younger horses and horses with access to stagnant water at pasture were found to be at significantly increased risk of IFEE. However, this study was conducted over a limited time-period in a relatively small number of IFEE cases. Based on the observations of the primary author, IFEE cases diagnosed in a single equine hospital appeared to present within a short time of each other and from similar geographic regions.

Currently, a lack of knowledge regarding the aetiology of IFEE lesions and risk factors for this specific form of intestinal disease prevents horses at increased risk from being identified and limits evidence-based information that can be given to owners and/or carers of horses about possible disease prevention. Based on a UK equine hospital population, the aims of the present study were to investigate temporal changes in IFEE risk, to ascertain the effect of age on the risk process and to examine for geographical variation in IFEE risk. We hypothesised that young horses would be at increased risk and that IFEE risk would vary geographically and exhibit some form of temporal (time-related) trend.

## Materials and Methods

### Study design and data collection

**Figure 1:**
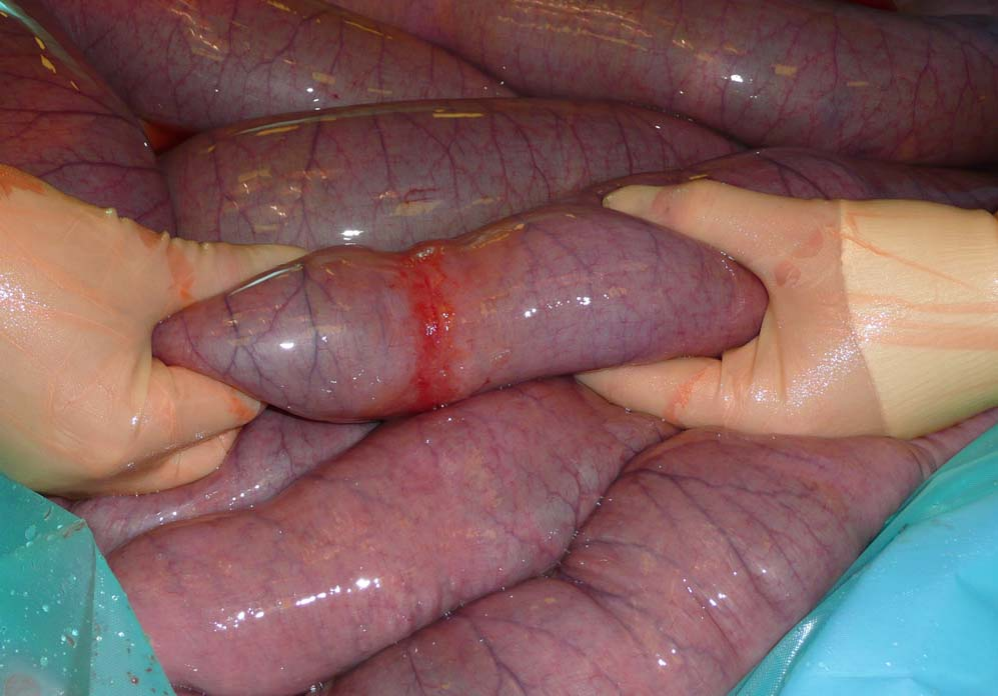
The typical visual appearance of an idiopathic focal eosinophilic enteritis IFEE lesion at laparotomy. The lesion is grossly hyperaemic and spans all or part of the circumference of the small intestine (small bowel). Palpably the tissues are markedly thickened at the site of the lesion and most commonly there is obstruction of ingesta proximal to the lesion(s) resulting in a simple obstruction of the bowel.

A nested case-control study design was utilised to determine whether age, time, season and geographical location had an effect on IFEE risk. Cases were all horses (defined as horses or ponies) in which a diagnosis of IFEE was made at exploratory laparotomy between 1^st^ July 2000 and 30^th^ June 2010 at the Philip Leverhulme Equine Hospital (PLEH), University of Liverpool. Diagnosis was based upon identification of the characteristic gross lesions at laparotomy (Figure 1) and where these lesions were considered to be the primary cause of abdominal pain. For each IFEE case, 10 controls were selected from those horses that had undergone exploratory laparotomy at the hospital at some point over the 10 year study period, the same period over which cases were collected. For all controls IFEE had been excluded as a cause of colic following exploration of the gastrointestinal tract. Simple random sampling was used, with all potential controls over the study period being equally likely to be selected; controls were not matched on any of the variables.

For each horse, the date of surgery and their age (in years) at the time of surgery were recorded. Georeferencing was based upon the unit postcode of each horse's premises at the time of diagnosis (where known) or that of the horse's owner/usual carer. For mapping and modelling purposes the postcode data were converted to ordinance survey ‘Eastings’ and ‘Northings’ using software available at www.streetmap.co.uk. This work was performed under approval from the University of Liverpool Veterinary Ethics Committee.

### Statistical analysis

Statistical analyses were performed using R software version 2.10.1 [34]. The R packages ‘splancs’ (version 1.8–2) and ‘mgcv’ (version 2.01–34) were used for point-mapping and for fitting generalised linear and generalised additive models, respectively. Statistical significance was set at *p* = 0.05.

Exploratory data analysis was conducted to investigate the form of the relationships, if any, between the horse's case-control status and its age and the date of surgery. Horse age was collapsed to form 11 age categories: 0–2, 2–4, …, 16–18, 18–21,>21 years and the date of surgery was considered in terms of the year: 2000, 2001, ...., 2010 and also the calendar month. Chi-squared tests were used to formally test for associations. A point map was used to depict the spatial locations of the cases and controls over the study region; the location of the PLEH and the UK coastline was added to the figure to aid interpretation.

### Statistical modelling

Using a logistic link function and Bernoulli data model, generalised linear models (GLMs) [35] and generalised additive models (GAMs) [36] were used to model the effects of horse age, time, season and geographical location on the logarithm of the odds, (*P*/(1–*P*)), of IFEE, where *P* is the probability of being a case. In a GLM the functional form of the relationship is parametric (such as linear, quadratic or sinusoidal) and is fixed in advance. In contrast, in a GAM the (smooth) functional form, known as *the smooth*, is unspecified (non-parametric) and is estimated as part of the model fit, thereby providing greater flexibility. Models including both parametric and smooth terms are said to be semi-parametric; all model sub-types (parametric, additive, and semi-parametric) can be fitted within the GAM modelling framework. Wood [37] provides a comprehensive guide to GAM models and their application using R.

Both parametric (GLM) and additive (GAM) model formulations were considered for each of the covariates, age and study time (in days from 1^st^ January 2001). The aim was to obtain the simplest functional form for each model term whilst also seeking to avoid model mis-specification. The contribution of the geographical location, *x* =  (*x*1, *x*2)  =  (Easting, Northing), to the log-odds was considered likely to be complex in nature and so a smooth (rather than parametric) functional form was considered for this spatial log-odds surface. Using the unbiased risk estimator (UBRE) score (generalised AIC) a forward selection procedure was used for model selection. This approach allowed for any confounding to be dealt with and provided a flexible framework for identification of the covariates that best and most parsimoniously explained the odds of being a case. Moreover, the resultant spatial smooth and associated standard error can be mapped over the study region, providing an estimate of ‘residual’ spatial variation in IFEE risk, adjusted for known confounders.

Since the cases and controls derive from the same sub-population and their IFEE status was not known on admission, it seemed reasonable to assume that the chance of being a case would not depend upon distance from hospital. However, to allow for any potential confounding effects of hospital location, the Euclidean distance from the hospital to each case and control location was computed and a *t*- test was used to check for any gross discrepancy in this so-called ‘hospital distance’ between cases and controls. Hospital distance was also included in the list of potential explanatory variables in the model.

### Uncertainty in location

Due to the fact that the owner/carer postcode may not represent the ‘true’ location of the horse at the time of development of disease, the robustness of the results to likely errors in the georeferencing was investigated. This was undertaken by examining the distribution of distances, *f*(*d*), between the ‘observed’ carer post-code and the ‘true’ location of each of *n* = 100 horses randomly selected from those admitted to the PLEH during 2011. These included horses who were admitted for the diagnosis and treatment of colic and of other conditions (but who would potentially have been admitted for colic had this occurred). The true location of each horse immediately prior to hospital admission and that of the owner/carer was ascertained by case records (which specifically recorded these details from 2011 onwards) or by subsequent telephone contact with the owner/carer where these details had not been fully recorded. These were considered to be a representative sample of horses in the present study and ensured accuracy in determining true horse and owner/carer location by minimising recall bias. Recall bias would have been a potential issue had this data been sought for the actual horses in the present study, due to time elapsed prior to analysis being performed.

Rather than attempt to model the distribution of distances parametrically, the non-parametric bootstrap approach was used [38]. For a given horse in our case-control study a discrepancy distance, d*, was sampled from the empirical distribution, *f(d*), and the horse was then “relocated” to a point at random on the circle of radius d* centred at the owner/carer's location, *x*. Repeating this procedure for all horses provided an alternative set of co-ordinates with which to conduct the spatial analyses. Creating several such sets of perturbed co-ordinates and repeating the analyses enabled the sensitivity of the results to likely errors in each horse's location to be determined. A total of five new (perturbed) case-control datasets were considered. For each bootstrapped data set the GAM model formulation in equation (1) was re-fitted providing five adjusted parameter sets and five adjusted surface estimates for comparison.

## Results

**Figure 2:**
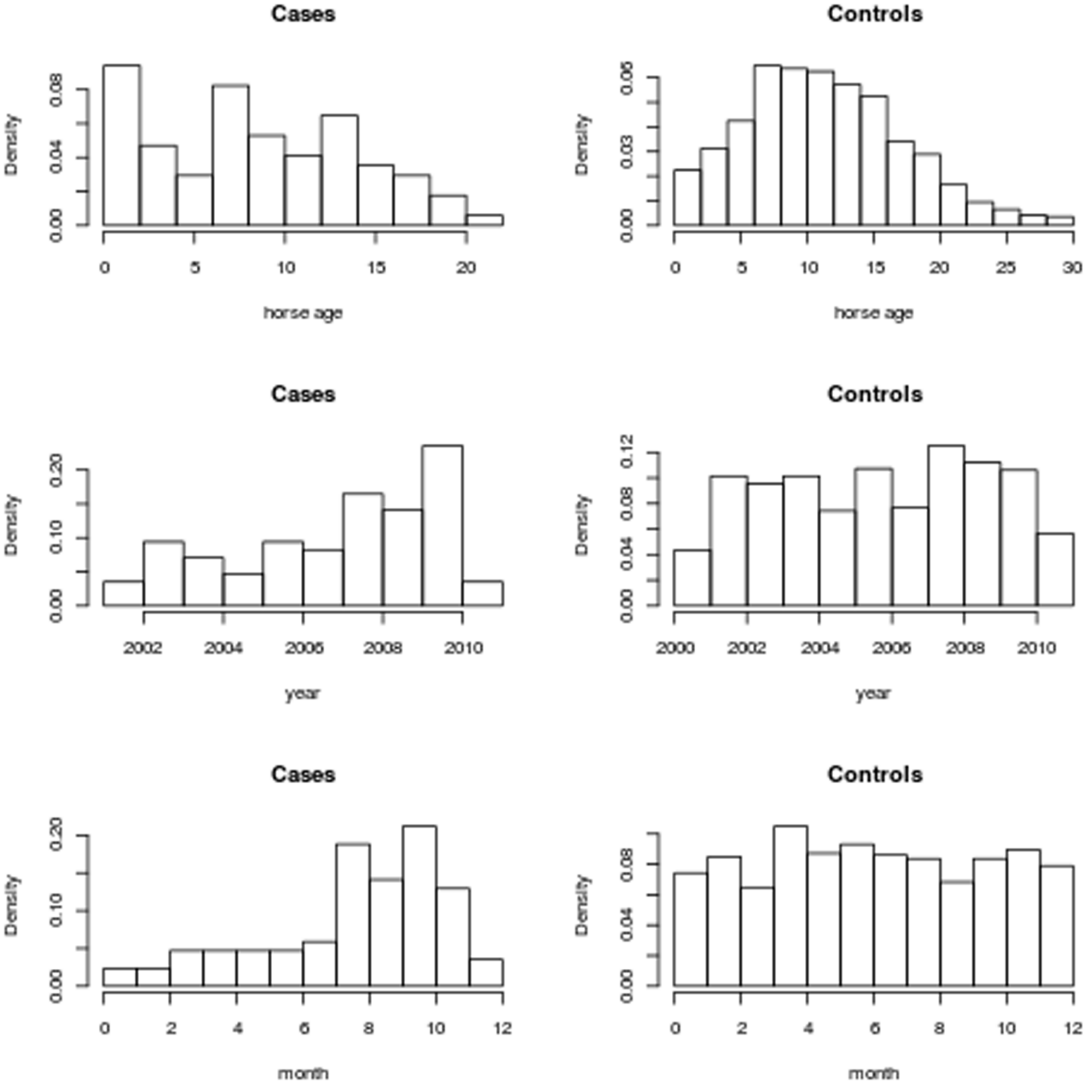
Frequency histograms of horse age, month and year. Histograms depicting the frequency distributions of the ages of the horses (top), the year of surgery (middle) and month of surgery (bottom) for cases (left) and controls (right). The frequencies for the years' 2000 and 2010 are circa half the size of those of the other years as the period of study spans July 2000 to June 2010.

Over the study period a total of 85 cases of IFEE were diagnosed. During the same period of time, 3116 colic cases were admitted to the hospital from which 850 randomly selected control (non-IFEE cases diagnosed at laparotomy) horses were drawn. Two randomly selected control horses were found to have been selected twice and so the duplicated data were excluded from further analysis. Histograms of the age, year and month at diagnosis for the case and control horses are shown in Figure 2. The relative frequency of younger horses was significantly greater in cases than amongst controls (p<0.0001). The frequencies of cases and of controls did not evolve in the same way over the ten year study period (p = 0.01). More specifically, the frequency of IFEE cases increased, whereas, the frequency of controls, on average, remained static. In addition, there was a significant difference in the month of diagnosis between cases and controls (p<0.0001). More specifically, the relative frequency of cases peaked between the months of July and October, whereas, that of the control horses was relatively consistent throughout each month. There was no significant difference between the mean distance of the cases from the PLEH and the mean distance of the controls (p = 0.439).

**Figure 3:**
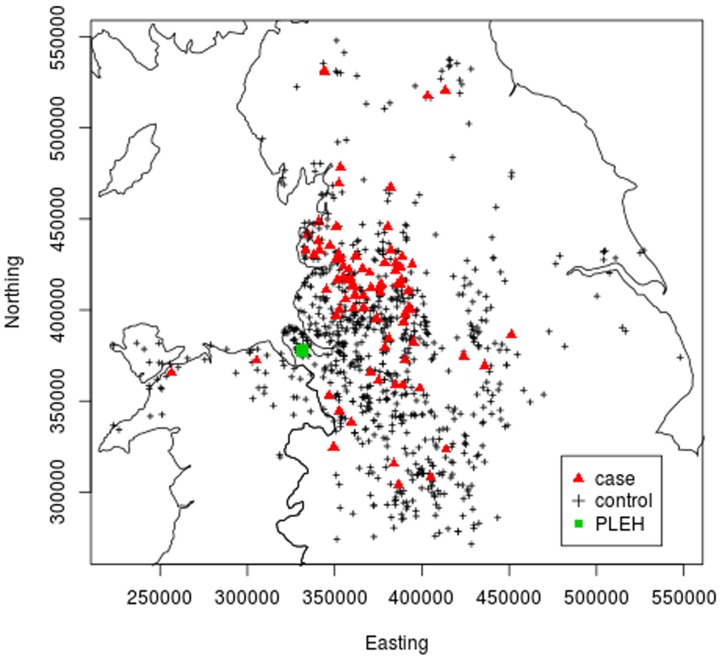
Geographic locations, *x* =  (*x*1, *x*2)  =  (Easting, Northing), of case and control horses. The point map depicts the geographical location of the 85 cases (red triangles) of idiopathic focal eosinophilic enteritis (IFEE) and 848 randomly selected controls (‘+’) in the present study. The green circle shows the location of the Philip Leverhulme Equine Hospital (PLEH).

The geographic locations of the owner/carer premises for the case and control horses are shown in Figure 3. This spatial distribution is representative of the hospital's referral population, with horses admitted to the PLEH for assessment of acute abdominal pain predominantly located in the North-West of England and some horses referred from neighbouring regions (i.e. North Wales, Midlands and the North-East of England). On initial, visual, inspection the majority of cases appeared to be located within the North-West region of the hospital's catchment area.

### Statistical modelling

Exploratory analysis of the data showed that a horse's age, its date of admission (in terms of both the year and the time of year), and its owner/carer location were all potentially important explanatory factors for predicting IFEE case-control status. The aim then was to find the most parsimonious model which best predicts the odds that a given horse is a case rather than a control. In formulating the model we wished to not only account for each of these potential factors, but also to consider different possible functional forms for each of them.

For the effect of a horse's age we considered parametric (including linear, quadratic and cubic terms) and also smooth model formulations. For the effect of geographical location we considered either linear effects or a spatial smooth. There are two potential problems with using a smooth to account for a seasonal effect and/or a gradual time-trend. Firstly, a seasonal smooth is unable to incorporate our understanding that the seasonal effect on the 31^st^ December (day 365) should be very similar to that on the 1^st^ January (day 1); this in turn leads to unnecessarily wide confidence intervals, especially in December and January. Secondly, a smooth over the whole study period would automatically try to account for both the long-term trend and the seasonal signal each year, with no recognition that this signal might be similar from year to year. Exploratory fits using such a smooth did indeed show the year on-year similarity. For the long term trend we therefore considered only a linear, a quadratic term, and a combination of the two. For the seasonal cycle we allowed for either annual or biannual cycles or the combination of the two using covariate terms of the form sin(2π*d*/365) and cos(2π*d*/365), and sin(*4πd*/365) and cos(4π*d*/365), where *d* is the day number since January 1st 2001.

When adjusted for ‘hospital distance’ there was no evidence of an effect due to hospital location; the UBRE score was (to 3 d.p.) unchanged on inclusion of this covariate. The final fitted model, using a forward selection procedure based upon minimising the UBRE score, was semi-parametric in form and consisted of parametric (linear and sinusoidal) terms in day since 1^st^ January 2001, *d*, and smooth terms of horse age (years) and geographical location and took the form: 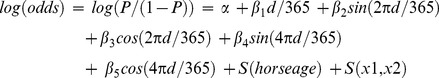
(1)where *x*1 and *x*2 are the Easting and Northing, respectively, of the owners/carer's location, and *S*(.) represents an arbitrary smooth function. In the case-control setting the intercept parameter, *α*, is of no interest and simply reflects the case-control sampling ratio.

**Figure 4:**
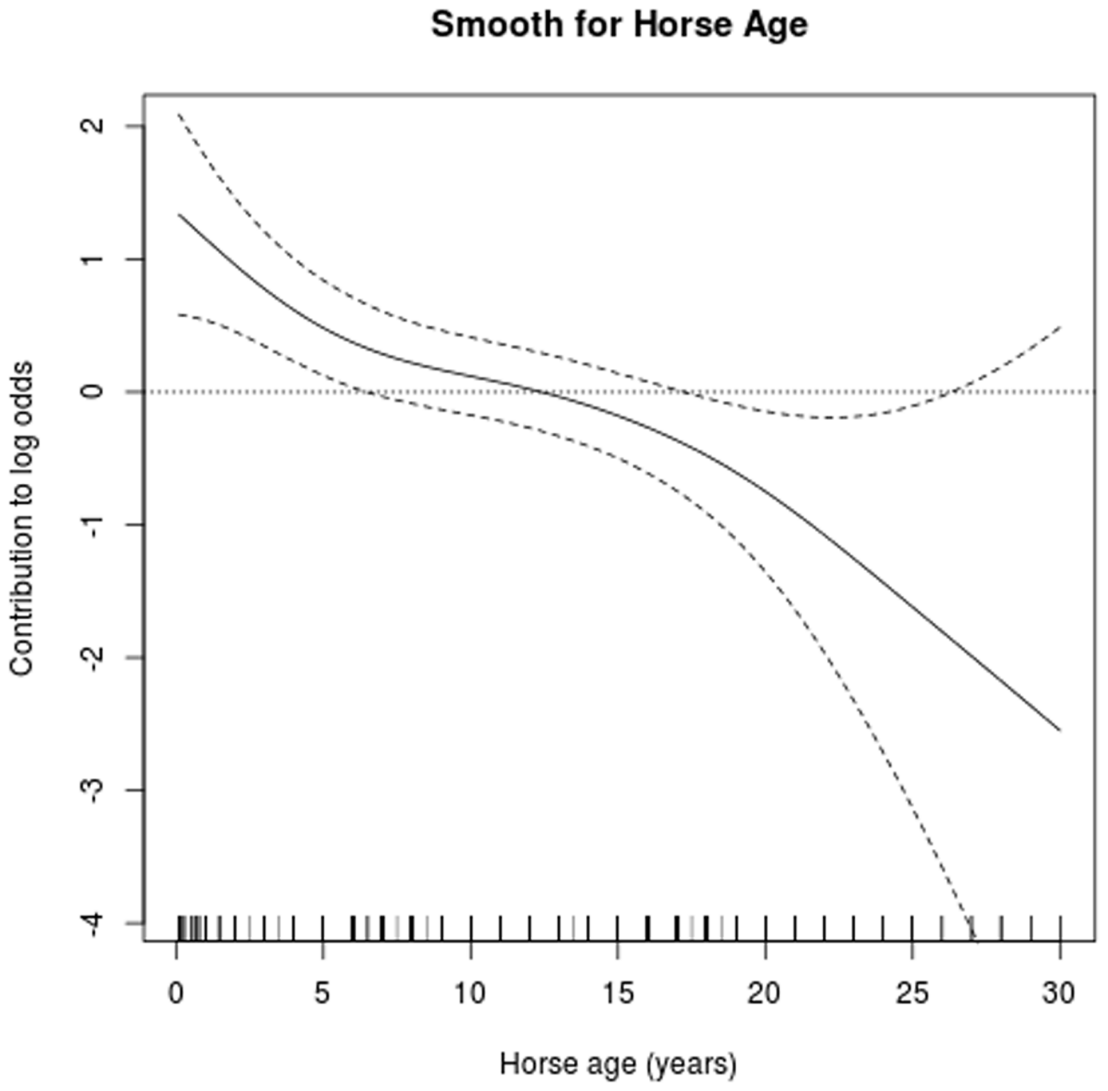
Graphical illustration of the estimated relationship (smooth) between the log odds of IFEE (‘Effect’) and age. The estimate derives from a Generalised Additive Model formulation; the smooth of horse age adjusted for seasonality (parametric), day in year (parametric) and the geographical location of the horse/owner (smooth). The dotted horizontal line is at log-odds  = 0.

**Figure 5:**
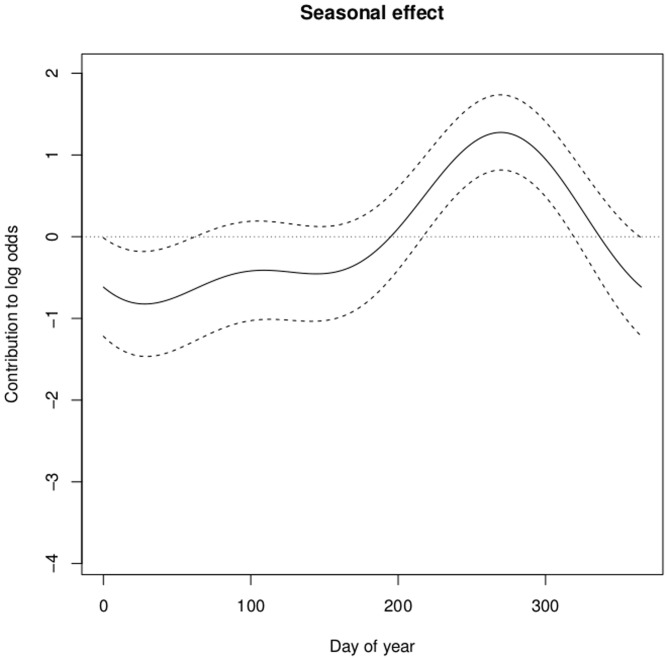
Graphical illustration of the estimated relationship between the log odds of IFEE (‘contribution to log odds’) and day of year. The seasonal estimate (parametric) derives from a Generalised Additive Model formulation and is adjusted for horse age (smooth) and the geographical location of the horse/horse owner (smooth). The dotted horizontal line is at log-odds  = 0.

**Figure 6:**
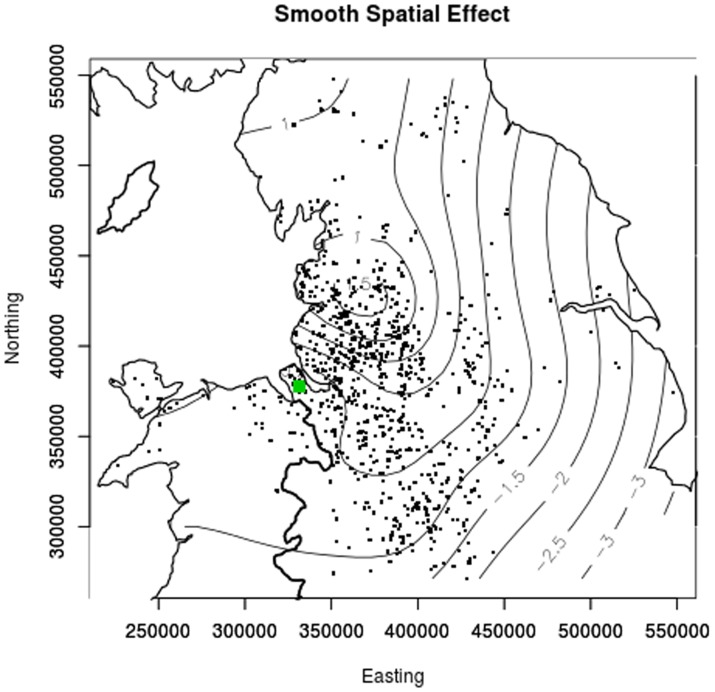
Contour plot of the smooth estimate of residual spatial variation in the log odds of IFEE. This is adjusted for day in year (parametric day of year and season effects) and horse age (smooth). The closed circular contours centred at Easting  = *x*1 = 370000 and Northing  = *x*2 = 4 25000 indicates an increase in the log odds in this region. A zero contour value corresponds to an odds of 1, positive values correspond to odds in excess of one and negative values to odds of less than one. The green circle shows the location of the Philip Leverhulme Equine Hospital (PLEH).

Parameter estimates and their *p*-values are provided in Table 1. In addition, the smooth terms for horse age and geographical location were both highly significant, with *p*-values of 0.0009 and 0.0001, respectively. The estimated smooth for a horse's age (Figure 4) showed that the risk of IFEE was greater in younger horses with a sharp decrease in risk over the first five years of life and a steadier decrease in risk thereafter. The estimated seasonal cycle (Figure 5) showed increased risk of IFEE between July and November (days 180–340) with the peak during the autumn months, whilst the parameter estimate for *β*_1_ in Table 1 indicates a year-on-year increase in the odds by a factor of approximately 1.18. The disease map depicting the residual spatial variation in the log odds of IFEE (Figure 6) indicated elevated IFEE risk in the North-West of the study region and reduced risk in the East and South of the region.

**Table 1:** Parameter estimates relating to the time (day in year) and seasonal effects for the final fitted model.

Parameter	Estimate	Standard Error	Z-value	P-value
β_1_	0.1604	0.0478	3.358	<0.001
β_2_	−0.8571	0.1955	−4.385	<0.0001
β_3_	−0.2036	0.2147	0.948	0.3431
β_4_	−0.0125	0.1911	−0.066	0.9477
β_5_	−0.4125	0.1960	−2.104	0.0353

### Uncertainty in location

The observed location discrepancy data, *f*(*d*), obtained from 100 randomly selected hospital admissions (comparing the ‘observed’ owner/carer locations with those of the ‘true’ location of each horse,) showed that all horses were kept within 27 km of their owner/carer. More specifically, 96 out of the 100 horses (96%) were located within 15 km and 77 (77%) were within 5 km of the owner/carer premises. Based upon those sampled, the overall mean distance between the ‘true’ horse premises and owner/carer location was 3.53 km for the study hospital.

On re-fitting the model in equation (1) to each of the five datasets, the parameter estimates, *β*, were materially unaltered (Table 2). This demonstrated that likely errors in the georeferencing of the horses' true location made no substantial difference to the parameter estimates, or their significance, in our final chosen model.

**Table 2:** Minimum and maximum values of the estimated parameters and associated *p*-values resulting from GAM model fits to the five sets of randomly permuted location data.

Parameter	Minimum Estimated Value	Maximum Estimated Value	Minimum P-value	Maximum P-value
β_1_	0.1601	0.1626	0.0006	0.0008
β_2_	−0.8688	−0.8444	<0.0001	<0.0001
β_3_	−0.2024	−0.2164	0.3113	0.3442
β_4_	−0.0210	−0.0035	0.9121	0.9582
β_5_	−0.4198	−0.4032	0.0315	0.0396

**Figure 7:**
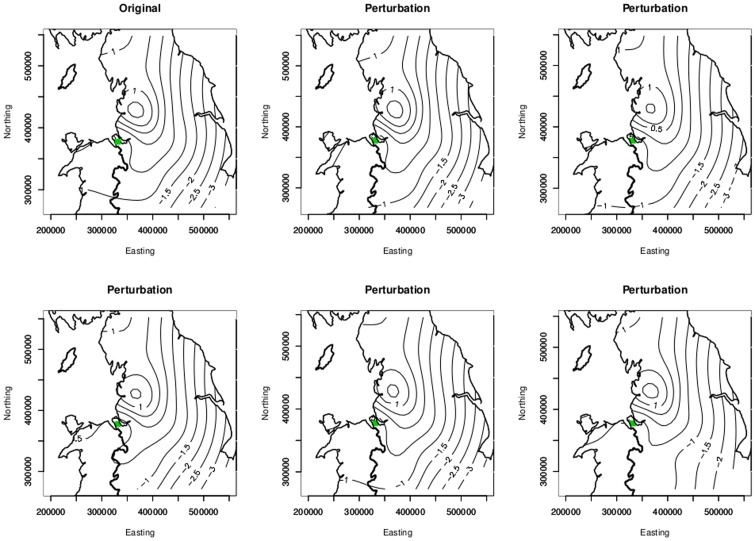
Contour plots of the smooth estimates of the residual spatial variation in the log odds of IFEE. These were obtained when using the observed location data of the owner/carer location (top left) and also when using each of the five perturbed sets of co-ordinates.

Figure 7 shows the contour plots of the surface obtained by using the recorded owner/carer location and by using each of the five perturbed sets of co-ordinates. Visual inspection of the plots showed no substantial differences from the original; all six plots clearly show that the risk is highest in the North-

West of the study area with lower risk to the East and South.

## Discussion

This is the first published study investigating the epidemiology of IFEE, a relatively new and important form of intestinal disease in horses that appears to be increasingly seen in some hospital populations and whose aetiology is currently unknown. This study provides important evidence-based information that may be used by veterinary clinicians to assist diagnosis of this condition and provides some clues regarding possible aetiological factors that require further investigation.

EGID have been increasingly recognised in the last 10–15 years as a clinically important and mechanistically baffling group of diseases in humans [22], [23]. This has some interesting parallels with IFEE in horses, with the present study confirming a consistent trend (increase) in numbers of cases seen in a UK equine hospital population over a 10 year period. The characteristic lesions seen in equine IFEE cases do not appear to occur commonly in humans or other animal species, but there are reports of focal lesions associated with marked eosinophilic infiltration of the small bowel in humans in small numbers of case reports in the literature [20], [39]. The clinical presentation of human eosinophilic gastroenteritis is related to the degree and area of the gastrointestinal tract affected. In the muscularis form, which is most analogous to IFEE lesions in horses, gastrointestinal obstructive symptoms may be seen and the serosal form is characterised by exudative ascites [21]. This is consistent with the clinical findings in affected horses. However, in horses IFEE lesions appear to be transient [13], [18] and recurrence is extremely rare [13], [18], with no need for ongoing dietary restriction and corticosteroid medication, which is the mainstay of therapy in humans [22] and in horses with the diffuse, chronic form of eosinophilic gastrointestinal disease [28]. Therefore there is little evidence within the scientific literature from other species regarding the possible aetiology of this condition in horses.

Historically there have been many anecdotal suggestions by veterinary surgeons and horse owners that equine colic has a seasonal pattern of occurrence. Scientific confirmation that there is seasonal variation in horses with colic presented to a referral hospital was provided by Archer et al. [40]. The latter study demonstrated not only that colic cases presented to a referral hospital had a biannual cyclical pattern (with peaks occurring in spring and autumn months) but also that some specific forms of colic had different temporal (seasonal) patterns. In the present study, we have demonstrated for the first time that the incidence rate of IFEE compared with the rate for all other types of colic has a seasonal pattern with a higher relative incidence rate in the late summer and autumn months. This is an important finding as this seasonal pattern differs from other types of colic previously investigated. In addition to assisting diagnosis of possible IFEE cases, this evidence may assist in ruling in or out potential aetiological factors. The seasonal pattern identified may implicate particular environmental conditions or other risk factors such as management practices that vary seasonally and to which affected horses may be more likely to have been exposed as potential contributing factors. Preliminary epidemiological investigations by Archer et al. [33] did not identify any managemental practices that increase the risk of IFEE in a multicentre study, although the study was of too low a power to detect relatively small differences in risk. However, in the same study, horses with access to stagnant water were found to be at increased risk of IFEE. It is therefore plausible that the months during which horses were identified to be at increased risk of IFEE in the present study may represent times of the year when wetter weather conditions may predominate and horses may have access to (and potentially ingest) stagnant water. Future research efforts therefore should consider investigating patterns of rainfall and analysis of water samples from pastures that affected horses have grazed on at the time of disease occurrence to determine whether a potential aetiologic agent is associated with these factors.

The present study also confirmed evidence of spatial variation in disease risk, with increased risk of IFEE in horses located in the North-West of the hospital catchment area (Lancashire region and surrounding areas) compared to regions in the South and East. There is no reason why there should be any selection bias in the location of cases or controls due to the fact that all the horses were referred for assessment of acute abdominal pain, the exact cause of which is often unknown based upon initial assessment on the horses' premises. Whilst the types of colic seen in different geographic regions is known to vary, this is often attributed to differences in equine populations regarding breed and use. Minimal research has been conducted to investigate spatial variation in risk of colic and specific forms of colic using appropriate statistical methods; *K*-function analysis has been utilised to investigate spatial and temporal variation in risk of equine dysautonomia (equine grass sickness), a disease that affects the equine gastrointestinal tract [41]. The results of the latter study provided important evidence-based information that could be used to assist diagnosis of cases and to identify horses in which preventive strategies should be targeted. Horse location is likely to be aggregated in space as some terrains may be better suited to keeping horses (e.g. less urbanised regions, suitable grazing areas). However, compared to control horses, IFEE cases in the present study were significantly more likely to occur in the North-West of the hospital catchment area. We have confirmed scientifically our suspicion that horses located in certain regions appear to be at increased risk of IFEE. This information can also be used to assist diagnosis (high risk horses) and to identify areas that require future research. Given that horse-level factors (e.g. age, breed) and managemental practices (e.g. feed types) within a relatively small geographical region of the UK are unlikely to vary according to this form of spatial distribution, it is plausible that environmental factors may play a role in the development of IFEE. Future research should therefore consider investigation of environmental factors such as soil types and pathogens that are associated with similar spatial patterns of disease in other species including humans.

The present study also demonstrated that younger horses were at increased risk of IFEE with the decrease in risk steepest over the age range of 0–5 years. This is consistent with the preliminary findings of Archer et al. [33] where horses in this same age range were found to be at increased risk of IFEE and with a similar pattern in disease risk. It is possible that presentation of a novel antigenic stimulus to the lumen of the gastrointestinal tract may play a role in IFEE development in horses. However, unlike the situation in humans where an allergic component is recognised and ongoing medical therapy may be required in the management of EGID to suppress abnormal immune response, it is rare for IFEE lesions to recur in affected horses (hospital unpublished data) nor is ongoing dietary restriction or corticosteroid therapy required to prevent recurrence of signs of abdominal pain.

One limitation of this study was that the exact location of horses could not be determined from hospital records as this was information not routinely obtained for horses admitted to the hospital during the time period under investigation. The home postcode of the carer of the horse at the time of diagnosis was, therefore, used as a proxy for the horse location, potentially introducing a degree of error. We therefore obtained an approximation to the distribution of distances between ‘recorded’ and ‘true’ locations using a sample of 100 horses seen at the hospital between 2011–2012. It was found that over three quarters of the owner/carers in our sample resided within 5 km of the horse itself. This result is consistent with the findings of a recent study [42] conducted in the UK investigating the size and spatial distribution of the horse population in the UK. More generally, the accuracy in and choice of, georeferencing is a generic problem in environmental epidemiology and further studies investigating spatial variation in disease risk should, of course, seek to accurately record horses' true location. By creating several additional data sets where the recorded locations were perturbed according to our distribution of distance between horse and owner/carer locations and fitting our model to each of these data sets we have provided a simple methodology that allows us to gauge the likely impact on our inferences of inaccuracies in location due to use of the carer's residence rather than the horse's actual location. Here there was strong evidence that our inferences are robust to these inaccuracies. It should be noted, however, that the results of the present study may only be applicable to this population of horses and geographic region.

The present study compared IFEE cases to surgically confirmed non-IFEE colic cases, and not to the general population of horses in the region. We recognise that use of hospital patients as controls does have some limitations [43] including the potential for cases and controls to have similar aetiological features. Because we could not exclude IFEE as a potential cause of disease (which may have been sub-clinical if mild in nature) without exploring the gastrointestinal tract, we consider that the selection of controls used was robust and avoided misclassification. Our use of a nested design reduces the potential for bias and whilst aetiological features may be shared, this study provides important information on how the case and control populations differ which is relevant to furthering our understanding of this form of gastrointestinal disease.

The results of the present study demonstrate that young horses are at increased risk of IFEE and that this condition exhibits seasonality, with increased risk between the months of July and November. In addition, we have shown that the odds of IFEE increased over the study period and also that horses located within certain geographical areas are at increased risk. These findings can be used by clinicians to assist diagnosis of this condition by recognising factors that place horses at increased risk of IFEE. In addition, these findings justify the need for further research investigating the aetiology of this type of intestinal disease in the horse, including investigation of environmental factors that may be associated with altered risk of IFEE.
